# Prefrontal Asymmetry during Cognitive Tasks and Its Relationship with Suicide Ideation in Major Depressive Disorder: An fNIRS Study

**DOI:** 10.3390/diagnostics9040193

**Published:** 2019-11-15

**Authors:** Seung Yeon Baik, Jeong-Youn Kim, Jongkwan Choi, Ji Yeong Baek, Yeonsoo Park, Yourim Kim, Minjee Jung, Seung-Hwan Lee

**Affiliations:** 1 Clinical Emotion and Cognition Research Laboratory, Inje University, Goyang 411-706, Korea; sybaik91@gmail.com (S.Y.B.);; 2 OBELAB Inc., Seoul 06211, Korea; 3 Department of Psychiatry, Inje University, Ilsan Paik Hospital, Goyang 411-706, Korea

**Keywords:** spectroscopy, near-infrared, prefrontal cortex, cognition, depression, suicide

## Abstract

Reduced oxygenation changes in the prefrontal cortex during cognitive tasks have been reported in major depressive disorder (MDD). However, prefrontal asymmetry during cognitive tasks and its relation to suicide ideations have been less frequently examined in patients with MDD. This study investigated prefrontal asymmetry and its moderating effect on the relationship between depression severity and suicidal ideation in MDD patients during cognitive tasks. Forty-two patients with MDD and 64 healthy controls (HCs) were assessed for changes in oxygenated and deoxygenated hemoglobin (Hb) in the prefrontal cortex using functional near-infrared spectroscopy (fNIRS) during the verbal fluency task (VFT), Stroop task, and two-back task. Depression, anxiety, and suicide ideation were measured through self-report questionnaires. Relatively smaller left oxy-Hb changes during VFT, but not during the Stroop or two-back tasks, were found in MDD patients compared with HCs. Furthermore, prefrontal asymmetry during VFT moderated the effect of depression severity on suicide ideation, and was significantly and positively correlated with suicide ideation in patients with MDD. Specifically, relatively greater left oxy-Hb changes were associated with greater suicide ideation. These findings suggest fNIRS-measured prefrontal asymmetry as a potential biomarker for MDD and for the assessment of suicidal risk in patients with MDD.

## 1. Introduction

### 1.1. Background

Depression is a leading cause of disability worldwide, with over 300 million individuals suffering from the disorder [1]. At its worst, depression can be life-threatening; around 50% of suicide attempters are diagnosed with depression [2]. Over the past few decades, a large body of research has investigated the biomarkers of depression to facilitate diagnosis and optimize treatment decisions. One emerging diagnostic tool for depression is functional near-infrared spectroscopy (fNIRS), an optical neuroimaging technology that uses near-infrared light to track changes in the concentration of oxygenated and deoxygenated hemoglobin (Hb) [3]. The use and development of fNIRS has increased tremendously in neuroscience and psychiatric research, and is expected to become a robust and reliable clinical tool (for review, see the work of [4]). In particular, the technique of fNIRS is suitable for measuring oxygenation changes in prefrontal areas associated with cognitive tasks [5,6,7].

With regard to depression, reduced Hb changes during cognitive activation have been suggested as potential biomarkers [8]. Previous studies found that patients with major depressive disorder (MDD) show reduced prefrontal oxygenation during cognitive tasks, such as verbal fluency [8,9,10], and working memory tasks [11,12]. However, there are mixed evidence regarding hemispheric asymmetry. Some studies found relatively reduced Hb in the left prefrontal regions [13,14], whereas others did not report hemispheric differences during cognitive tasks in depressed individuals [15,16,17]. Nonetheless, previous studies suggest a significant association between prefrontal asymmetry and cognitive function in depression. For instance, a recent study discovered that the trait approach and avoidance motivation were associated with activation of the left and right prefrontal cortex, respectively, during executive functioning [18]. Likewise, depressed individuals who are characterized by avoidance motivation would show reduced left frontal activation during cognitive tasks. Other studies also suggested that deficits in executive functions in depression, such as the initiation and strategic use of information, may be because of the failure of left prefrontal cortex activation [19,20]. Moreover, relatively reduced left prefrontal activation (i.e., more electroencephalogram (EEG) alpha activity) during rest has been reported to be a trait marker of depression [21].

Importantly, comorbidity and different methodologies should be considered when studying prefrontal asymmetry. Depression is often associated with anxiety, which may show a pattern opposite to that of frontal asymmetry found in depression (i.e., relatively greater left frontal activity) [22,23]. In addition, frontal asymmetry may differ according to the presence of suicide ideation [24]. Methods to assess prefrontal asymmetry (e.g., hemisphere as a two-level factor in statistical analysis versus direct measurement using lateral index) and the running time of the applied cognitive test may affect the results as well. For example, in a verbal fluency task (VFT), a shorter duration trial (e.g., 30 s versus 60 s) may be more sensitive to the left frontal function, as it requires more frequent shifting among phonemes, and thus more initiation [25].

Meanwhile, suicide ideation is one of the symptoms of depression that is associated with cognitive rigidity and executive functioning deficits related to impairments of the frontal lobe [26,27,28]. Among depressed individuals, the pattern of prefrontal asymmetry was associated differently with suicide ideation. Suicide ideation was related to relatively increased activity in the left frontal regions of the brain, and patients with major depressive disorder (MDD) with suicide ideation showed greater left activity than patients without suicide ideation [24,29]. An imaging study also revealed a relative hypometabolism of the right dorsolateral cortex and hypermetabolism of the left ventromedial cortex in patients with MDD and a history of suicide attempts [30]. Moreover, smaller hemodynamic changes were found in the right prefrontal cortex in patients with MDD with suicide ideation compared with those without [31]. While these findings suggest that prefrontal asymmetry contributes to suicidality, the role of prefrontal asymmetry in linking depression to suicide ideation is expected to help identify depressed individuals with suicide risk. Also, the results of the previous studies need to be replicated owing to the limited number of studies and conflicting reports from others [32,33].

### 1.2. Aim of this Study

The present study aimed to investigate prefrontal asymmetry during cognitive tasks in patients with MDD, using fNIRS. We hypothesized that patients with MDD would show relatively reduced Hb in the left prefrontal cortex compared with healthy controls (HCs). We also hypothesized that relatively greater Hb levels in the left prefrontal cortex would be associated with suicide ideation and moderate the effect of depression severity on suicide ideation among patients with MDD.

## 2. Materials and Methods

### 2.1. Participants

A total of 125 participants (53 Patients with MDD and 72 HCs) between the ages of 19 and 65 from Ilsan Paik Hospital and the local community were included in this study. Participants with sensory impairments, epilepsy, and brain injury were excluded. In addition, we excluded patients with MDD with a history of psychosis symptoms and HCs with present or prior diagnosis of any type of mental disorder. MDD was diagnosed according to the Diagnostic and Statistical Manual of Mental Disorders, Fifth Edition [34], by a board-certified psychiatrist. Patients with MDD and HCs were matched with respect to age and sex.

One participant dropped out during the experiment, and eight participants were excluded from analysis owing to missing data. In addition, data exceeding ± 3 SD from the mean asymmetry index of each task (2 for Stroop task, 3 for VFT, and 5 for two-back task) were considered outliers and were excluded from analysis. Thus, the final sample consisted of 106 participants, which included 42 patients with MDD and 64 HCs.

All procedures were approved by the Institutional Review Board of Inje University Ilsan Paik Hospital (2017-10-013). Informed consent was obtained from all participants prior to study enrollment.

### 2.2. Psychological Measures

Depression severity was assessed using the Korean version of the Beck’s depression inventory-II (BDI-II), which is composed of 21 items rated on a four-point scale. This self-reported inventory showed a high Cronbach’s alpha (α = 0.85) and test–retest reliability (*r* = 0.75) [35]. The internal consistency of the current sample was α = 0.95. Anxiety severity was measured using the Korean version of the State-trait anxiety inventory, a self-reported inventory consisting of 20 items that assess state anxiety and 20 items that assess trait anxiety. The state anxiety section (SAI) was used for the current study. The internal consistencies of the Korean version of SAI and the current sample were α = 0.91 [36] and α = 0.96, respectively.

Finally, suicide ideation was assessed using the suicide item (item 3) of Hamilton’s depression rating scale (HAM-D) (1960) [31,37,38]. Item 3 assesses the level of suicide ideation and is rated on a 0–4 scale (0 = absent, 1 = feels life is not worth living, 2 = wishes to be dead or any thought of possible death to self, 3 = suicidal ideas or gesture, 4 = suicide attempts).

### 2.3. Cognitive Tasks

Hb changes were measured during three different cognitive tasks: VFT, Stroop task, and two-back task. These tasks were chosen as they measure executive functioning, which is a kind of cognitive deficit associated mostly with depression [39,40,41] and frontal asymmetry [42].

**Figure 1:**
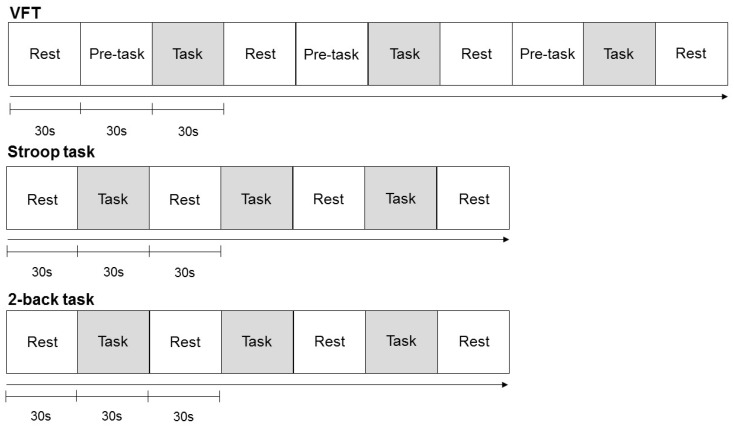
Cognitive task protocol used for the near-infrared spectroscopy (NIRSIT) system. Note: VFT = verbal fluency task.

The changes in Hb were measured while the participants performed all three cognitive tasks. The participants were seated in a comfortable chair and told to relax and avoid movements in order to minimize artifacts. They went through a practice session of the cognitive tasks (60–120 s per task) before the formal experiment. Each task was composed of three blocks with a 30 s resting period between the blocks (Figure 1). The tasks were designed using E-prime 2.0. Participants received a compensation of $50 if they properly completed the whole experiment.

#### 2.3.1. Verbal Fluency Task

VFT involves generating as many words that begin with a specified phoneme as possible within a certain time frame [43]. Each block of the VFT consisted of two parts: a 30 s pre-task baseline and the 30 s VFT task. During the pre-task, the participants were instructed to consecutively repeat the Korean consonants “g”, “n”, “d”, and “r” out loud. During the VFT task period, the participants were instructed to produce as many words beginning with a designated consonant as possible, while avoiding homonyms and proper nouns. The designated consonant was randomly selected from the eight consonants “g”, “n”, “d”, “r”, “m”, “b”, “s”, and “h”. All responses were recorded.

#### 2.3.2. Korean Stroop Task

A Korean version of the color-word Stroop task was administered to measure selective attention [44]. For each trial, two rows of words were displayed on a black screen. On the top row, a word appeared in one of the seven different colors, that is, “red”, “blue”, “yellow”, “gray”, “orange”, “pink”, or “green”, different from the one indicated by the word’s meaning (e.g., the word ”red” was written in ink color green). On the bottom row, two different words for colors were written in white on the left and right side. The ink color and the word stimulus were presented randomly among the seven colors. The participants had to decide which of the two colors at the bottom corresponded to the ink color of the word displayed on the top row by pressing the left and right arrow buttons on the keyboard (e.g., the left arrow was pressed to choose the word on the left) as quickly and accurately as possible. The next trial only started after the participants responded to this. The number of trials presented per block depended on the subject’s response speed.

#### 2.3.3. N-Back Task (Two-Back Task)

A two-back task with number stimuli was used to assess working memory [45]. During this task, the participants viewed numbers between 1 and 5 that successively appeared on the screen one at a time. Each number appeared on the screen for 1 s, with a 0.5 s interval between the trials. The numbers appeared in black against a white background, in the middle of the screen. The participants had to identify the number (i.e., the target) that corresponded to the number presented on the screen 2 trials earlier by pressing the left button of the mouse. The total number of trials per block was 20, and the target stimuli appeared in 40% of the trials.

### 2.4. fNIRS

A high density NIRS device (NIRSIT; OBELAB, Seoul, Korea) measured the relative changes in oxy-Hb and deoxy-Hb. The sensor array was comprised of 24 dual-wavelength laser diodes (780/850 nm) and 32 photo detectors separated by a 1.5 cm unit distance [46]. A 3 cm distance separated the laser and detector pairs at 48 sensing areas, and the optical signal variation of each channel was sampled at 8.138 Hz. The threshold signal-to-noise ratio was 30 dB, and it was used to qualify the noise of the detected channels after band-pass filtering from 0.005 to 0.1 Hz and remove the slow drift of physiological noise and environmental noise. Relative hemodynamic changes in each channel during each trial of each task were extracted using the Modified Beer Lambert Law (MBLL) [47]. The baseline value of relative change was defined as the averaged value from −5 to 0 s before the start of each task period. The multiple trial results are block averaged individually, and grand averaging was applied to extract the representative mapping result from each group.

Prefrontal asymmetry was defined as a lateral index, which measured the difference in Hb concentration changes between the left and right hemispheres divided by their sum: (left Hb − right Hb)/(left Hb + right Hb). A positive value indicates greater Hb in the left hemisphere, whereas a negative value indicates greater Hb in the right hemisphere.

### 2.5. Statistical Analyses

Behavioral data and the asymmetry index were compared between the groups using one-way analysis of covariance (ANCOVA). In addition to education, which significantly differed between the groups, age, sex, SAI, and item 3 of HAM-D3 were included as covariates because of their potential effects on hemodynamic responses and frontal asymmetry [23,24,48,49]. Also, correlation analyses were performed to examine the relationships of the asymmetry index of the three tasks with psychological and behavioral measures. Bootstrap resampling (*n* = 5000) was used to correct multiple correlations [50]. Finally, the moderating role of prefrontal asymmetry on the link between depression and suicide ideation was assessed using SPSS macro PROCESS v3.1.

The significance level was set at *p* < 0.05 (two-tailed). Statistical analyses were performed using SPSS 21 (SPSS, INC., Chicago, IL, USA).

## 3. Results

The analysis of deoxy-Hb was not significant; therefore, we focused on the oxy-Hb results. Oxy-Hb concentration has been suggested to better reflect cortical activity and be more strongly correlated with blood oxygenation level dependent (BOLD) signals measured by fMRI [51,52].

### 3.1. Demographics and Other Characteristics

**Figure 2:**
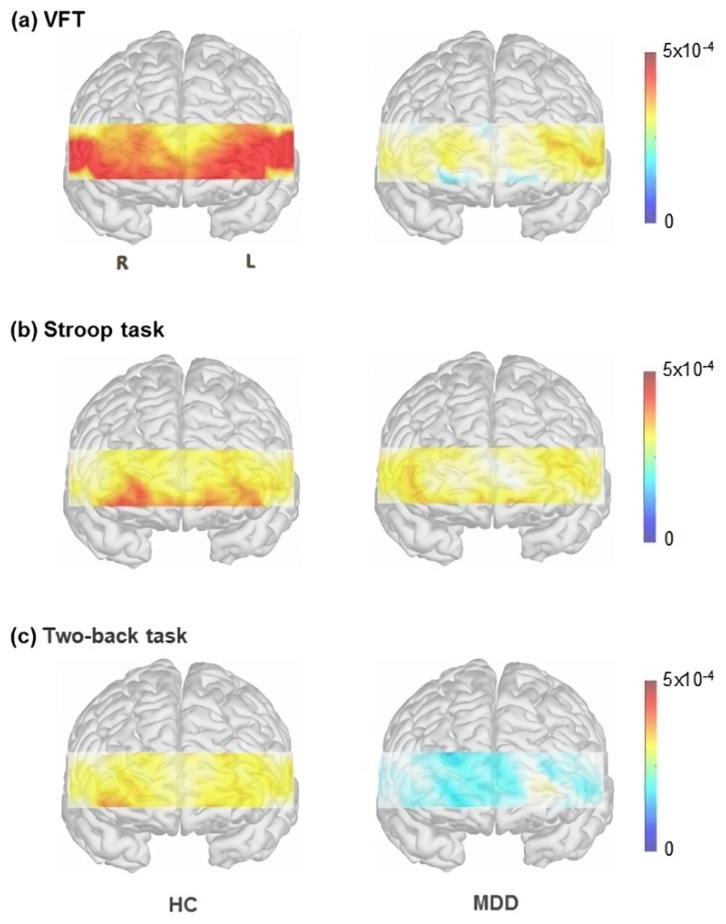
Oxygenated hemoglobin changes in patients with MDD and HCs during (**a**) Stroop task; (**b**) VFT; and (**c**) two-back task. Note: MDD = major depressive disorder, HCs = healthy controls; VFT = verbal fluency task.

Demographic information and scores of psychological scales are presented in Table 1. Patients with MDD had significantly fewer years of education (*p* = 0.020) and higher Beck’s depression inventory-II (BDI-II) (*p* < 0.001), State anxiety inventory (SAI) (*p* < 0.001), and Hamilton’s depression rating scale (HAM-D) (*p* < 0.001) scores compared with HCs. Overall oxy-Hb changes during the cognitive tasks are presented in Figure 2.

### 3.2. Behavioral Analysis

There were no significant group differences in the average number of words generated in VFT or the accuracy and reaction time for the Stroop task and two-back task (Table 2).

### 3.3. Prefrontal Asymmetry

#### 3.3.1. Group Differences in Prefrontal Asymmetry

**Figure 3:**
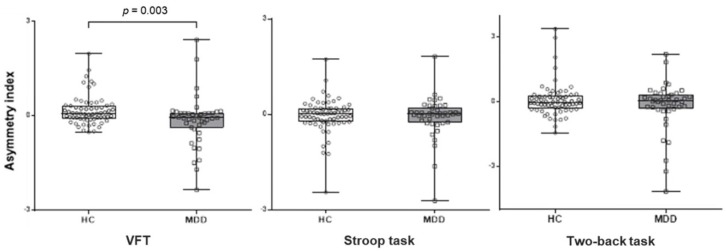
Mean asymmetry index during VFT, Stroop, and two-back tasks in patients with MDD and HCs. Asymmetry index was measured using the lateral index: (left oxy-Hb − right oxy-Hb)/(left oxy − Hb + right oxy-Hb). Note: MDD = major depressive disorder; HCs = healthy controls; VFT = verbal fluency task.

For VFT, one-way ANCOVA revealed a significant main effect of the group on prefrontal asymmetry for oxy-Hb (F (1, 97) = 9.12, *p* = 0.003). The mean asymmetry index for oxy-Hb was significantly lower in patients with MDD (M = −0.13, SD = 0.46) compared with the HCs (M = 0.17, SD = 0.80). In other words, mean oxy-Hb concentration changes were relatively lower in the left hemisphere compared with the right hemisphere in patients with MDD, whereas the opposite pattern was found in HC (Figure 3). In order to examine the effects of age and sex on asymmetry, we performed an additional ANCOVA modeling with age and sex, and did not find a significant effect of age (F (1, 102) = 0.667, *p* = 0.192) or sex (F (1, 102) = 0.119, *p* = 0.581) on the group differences in oxy-Hb changes during VFT. No significant results were found for either the Stroop or two-back tasks.

For deoxy-Hb data, there were no significant group differences in the mean asymmetry index for VFT (F (1, 97) = 4.215, *p* = 0.090), Stroop (F (1, 97) = 3.470, *p* = 0.066), and two-back tasks (F (1, 97) = 9.12, *p* = 0.060).

#### 3.3.2. Correlation between Asymmetry Index and Psychological Measures

For patients with MDD, there was a significant positive correlation between the asymmetry index for VFT and the suicide item (item 3) of HAM-D (r = 0.313, *p* = 0.046). For HC, we found a significant negative correlation between the asymmetry index for the Stroop task and SAI (r = −0.337, *p* = 0.006). There were no other significant correlations among BDI-II, SAI, item 3 of HAM-D, and asymmetry index of the three tasks. For all participants (patients with MDD + HC), there were no significant correlations.

#### 3.3.3. Correlation between Asymmetry Index and Behavioral Measures

There was a significant positive correlation between the asymmetry index and response accuracy for the two-back task (r = 0.282, *p* = 0.007) in MDD patients. However, there were no significant correlations between asymmetry index and behavioral measures for the VFT and Stroop task.

### 3.4. Moderation Effect

**Figure 4:**
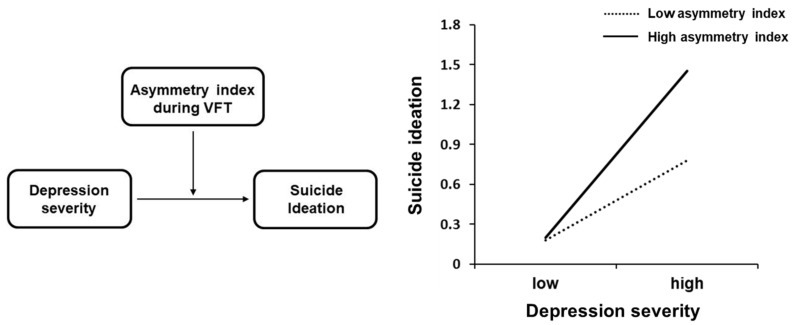
Moderating effect of prefrontal asymmetry index on the relationship between depression and suicide ideation in patients with major depressive disorder (MDD). Note: VFT = verbal fluency task.

For VFT, the moderation model suggested a significant interaction effect of prefrontal asymmetry for oxy-Hb and depression severity on suicide ideation (Table 3, Figure 4). The variance increase due to the interaction effect was R^2^ = 0.11. The 95% confidence interval (CI) did not cross the value zero, CI [0.009, 0.039]. Furthermore, the conditional indirect effect revealed that the relationship between depression severity and suicide ideation was moderated by prefrontal asymmetry at all levels (i.e., high, average, low) of the moderator. However, the strength of moderation was stronger at one SD above (i.e., greater left concentration), compared with one SD below (i.e., reduced left concentration) the mean asymmetry index. No significant interaction effects were found for the Stroop or two-back tasks. In addition, there were no significant interaction effects between depression severity and the mean asymmetry index for deoxy-Hb in VFT (*p* = 0.363), Stroop (*p* = 0.112), and two-back tasks (*p* = 0.163).

## 4. Discussion

This study investigated prefrontal hemispheric asymmetry in patients with MDD by measuring oxy-Hb concentration changes using fNIRS during cognitive tasks and its moderating role in the relationship between depression severity and suicide ideation. Our results indicated that patients with MDD have relatively reduced left prefrontal oxy-Hb changes during VFT compared with HCs. Furthermore, prefrontal asymmetry moderated the relationship between depression severity and suicide ideation. Specifically, the effect of depression severity on suicide ideation was stronger when the left prefrontal activation was higher.

The findings from the present study suggest that relatively reduced left prefrontal activity during VFT distinguishes patients with MDD from HCs. These results are consistent with that of previous studies that found hypoactivity to be most prominent in the left frontal lobe in depressed individuals during VFT. For instance, Ohta et al. demonstrated that attenuated prefrontal activity in patients with MDD was most pronounced in the left medial inferior lobe during VFT. Another NIRS study indicated reduced activity of the left frontal cortex in patients with mandatory depressive symptoms (depressed mood or loss of interest or pleasure), but not in remitted patients with residual symptoms during VFT [14]. Moreover, positron emission tomography (PET) [53,54] and EEG studies [21,55] have shown reduced activity in the left frontal cortex in patients with MDD.

In contrast, other studies found bilateral hypoactivity without any difference in the activation levels between the hemispheres [16,17,56] and even a similar level of activation with HCs in both hemispheres [57] in patients with MDD. The different results may be the result of several methodological differences. Most of these studies did not take suicide ideation or anxiety symptoms into account, whereas these were controlled as covariates in the present study. Furthermore, unlike most studies that statistically investigated the effect of hemisphere using repeated measures ANOVA, this study computed the asymmetry index to observe the relative difference between hemispheres. Lateral asymmetry index has several advantages such as high internal consistency [58] and the avoidance of individual differences like skull thickness [59,60]. Lastly, the duration of each VFT trial was shorter in the current study (30 s) compared with previous studies (mostly 60 s). A shorter time constraint may indicate greater initiation, as it requires more shifts from one phoneme to another, which is suggested to be associated with left frontal lobe activity [25,61].

In addition, there was no significant correlation between total word count during VFT and the asymmetry index. This indicates that VFT performance is unrelated to the oxygenation changes, as also reported by previous studies [62,63,64]. Thus, it is unlikely that the group differences in oxy-Hb changes found in the present study contributed to the performance of VFT per se.

The second major finding of this study was the moderating effect of prefrontal asymmetry on the link between depression severity and suicide ideation. We found that the effect of depression on suicide ideation was stronger when there was relatively greater left prefrontal asymmetry. The correlational analysis also revealed that a relatively higher left oxy-Hb concentration was associated with greater suicide ideation, further supporting the moderation effect. These results are in line with those of previous studies that found relatively greater left frontal activity in individuals with suicide ideation [24,29,31]. The moderation effect may be a result of the involvement of relatively greater left or reduced right prefrontal activity with behaviors like impulsivity, a characteristic that can distinguish between suicidal and non-suicidal patients with MDD [65,66,67]. Gable et al. [68] also proposed that the right frontal cortex is associated with the regulation and supervision of the motivational systems; thus, its hypoactivity indicates deficits of inhibitory control. Therefore, relatively greater left prefrontal activation compared with right may reflect reduced cognitive control or greater impulsivity, which may lead to greater suicidal ideation among patients with MDD. Nonetheless, it is also plausible that the effect of prefrontal asymmetry reflects the processing of VFT, unrelated to aspects of cognitive control. As there is a scarcity of literature on frontal asymmetry and suicide ideation, future studies are necessary to examine the underlying mechanisms of the relationship between frontal asymmetry and suicide ideation among patients with MDD.

Lastly, all significant findings in our study were specific to VFT, and insignificant results were only obtained in the Stroop and two-back tasks. This may indicate that the relatively attenuated left prefrontal activation in patients with MDD is associated with distinct characteristics of VFT. In fact, VFT has been shown to be largely associated with left prefrontal regions [69,70], whereas relatively few studies have examined hemispheric differences in the Stroop and two-back tasks [71,72,73], leading to ambiguous results. Moreover, several researchers [25,74] have suggested that VFT may be particularly sensitive to depression owing to the overlap between the cognitive demands of VFT and the cognitive deficits associated with depression, such as initiation, sustained attention, retrieval, and persistence.

There are several limitations to this study. First, extracerebral hemodynamics caused by physiological changes were not separated from measured signals, as the fNIRS device used in this study does not have a short distance channel under 1 cm [75]. Yet, previous studies like that of Takizawa et al. (2014) showed that the hemodynamics parameter derived from averaging during each task period without short channel separation can be used for assessing patients’ differential depressive state. Second, although we attempted to statistically control anxiety severity, some participants had anxiety symptoms. Various anxiety domains, such as anxious apprehension and anxious arousal, have been suggested to show different patterns of frontal asymmetry [22,23]. Third, most of the patients were under medication. The results of studies into the effects of antidepressants on NIRS signaling have been mixed, with some studies reporting no or minor effects [76,77,78] and others reporting major effects of certain drugs on prefrontal NIRS signals [79,80,81]. In the present study, we found no significant relationship between medication status and oxy-Hb signals.

## 5. Conclusions

The present study demonstrated that patients with MDD exhibit relatively reduced left prefrontal oxy-Hb changes during VFT compared with HCs. Also, there was a moderating effect of prefrontal asymmetry on the link between depression severity and suicide ideation. These results suggest that prefrontal asymmetry, measured by fNIRS, is a potential biomarker for MDD diagnosis and for identifying suicidal risk in patients with MDD. Future studies could assess how factors like motivation and cognitive control relate to prefrontal asymmetry during cognitive tasks in depression.

## Figures and Tables

**Table 1:** Demographic characteristics of participants.

	Patients with MDD (*n* = 42)	HCs (*n* = 64)	*p*
**Age**	37.62 ± 14.36	33.42 ± 12.57	0.115
**Sex**			0.685
**Male**	17	24	
**Female**	34	41	
**Education (years)**	11.62 ± 2.71	12.98 ± 3.03	0.020
**BDI-II**	22.21 ± 13.74	8.51 ± 7.02	<0.001
**SAI**	49.95 ± 13.34	34.11 ± 8.07	<0.001
**HAM-D**	20.31 ± 11.14	4.84 ± 5.06	<0.001

Note: MDD = major depressive disorder; HCs = healthy controls; BDI-II = Beck’s depression inventory-II; SAI = State anxiety inventory; HAM-D = Hamilton depression rating scale.

**Table 2:** Behavioral analyses of patients with MDD and HCs with age, sex, education, item 3 of HAM-D, and SAI as covariates.

	Patients with MDD	HC	*F*	*p*
**VFT**				
Word count	20.56 ± 8.50	24.67 ± 6.99	0.149	0.700
**Stroop task**				
Accuracy	0.89 ± 0.11	0.93 ± 0.06	0.287	0.594
RT (ms)	1815.82 ± 1055.01	1345.27 ± 690.72	1.377	0.243
**Two-back task**				
Accuracy	0.78 ± 0.10	0.84 ± 0.10	0.364	0.548
RT (ms)	585.44 ± 89.79	593.42 ± 90.66	1.105	0.322

Note: MDD = major depressive disorder; HCs = healthy controls; VFT = verbal fluency task; HAM-D = Hamilton depression rating scale; SAI = State anxiety inventory; RT = reaction time; Bold text in the first column indicates the type of cognitive task.

**Table 3:** Moderation analysis of the relationship between depression severity and suicide ideation by prefrontal asymmetry during VFT among patients with MDD.

	*B*	*se*	*t*	*p*	*LLCI*	*ULCI*	*R* ^2^	Δ*R*^2^	*F*
Constant	−1.036	1.071	−0.969	0.338	−3.202	1.127	0.442		6.332 ***
BDI	0.027	0.019	1.465	0.151	−0.010	0.065			
VFT asymmetry	−0.134	0.193	−0.696	0.491	−0.523	0.256			
Sex	0.145	0.497	0.291	0.773	−0.860	1.149			
SAI	0.028	0.016	1.773	0.084	−0.004	0.060			
BDI * VFT asymmetry	0.027	0.012	2.276	0.028	0.0031	0.053		0.072	5.176 *
Conditional Indirect effect			**VFT asymmetry**		**Effect**	**Boot *LLCI***	**Boot *ULCI***		
	Mean − 1 *SD*		−0.931		0.023	0.004	0.042		
	Mean		−0.130		0.042	0.026	0.057		
	Mean + 1 *SD*		0.670		0.061	0.040	0.081		

Note: VFT = verbal fluency task; MDD = major depressive disorder; LLCI: lower levels for confidence interval; ULCI: upper levels for confidence interval, * *p* < 0.05, *** *p* < 0.001.

## Abbreviations

MDD	Major depressive disorder
HC	Healthy control
Hb	Hemoglobin
fNIRS	Functional near-infrared spectroscopy
VFT	Verbal fluency task
BDI-II	Beck’s depression inventory-II
SAI	State anxiety inventory
HAM-D	Hamilton’s depression rating scale
EEG	Electroencephalogram
